# Efficient Interference Estimation with Accuracy Control for Data-Driven Resource Allocation in Cloud-RAN †

**DOI:** 10.3390/s18093000

**Published:** 2018-09-07

**Authors:** Yanchao Zhao, Jie Wu, Wenzhong Li, Sanglu Lu

**Affiliations:** 1 College of Computer Science and Technology, Nanjing University of Aeronautics and Astronautics, Nanjing 211106, China; 2 State Key Laboratory for Novel Software Technology, Nanjing University, Nanjing 210023, China; lwz@nju.edu.cn (W.L.); sanglu@nju.edu.cn (S.L.); 3 Collaborative Innovation Center of Novel Software Technology and Industrialization, Nanjing 210023, China; 4 Department of Computer and Information Sciences, Temple University, Philadelphia, PA 19121, USA; jiewu@temple.edu

**Keywords:** cloud-RAN, edge computing, resource allocation, interference measurement, modeling

## Abstract

The emerging edge computing paradigm has given rise to a new promising mobile network architecture, which can address a number of challenges that the operators are facing while trying to support growing end user’s needs by shifting the computation from the base station to the edge cloud computing facilities. With such powerfully computational power, traditional unpractical resource allocation algorithms could be feasible. However, even with near optimal algorithms, the allocation result could still be far from optimal due to the inaccurate modeling of interference among sensor nodes. Such a dilemma calls for a measurement data-driven resource allocation to improve the total capacity. Meanwhile, the measurement process of inter-nodes’ interference could be tedious, time-consuming and have low accuracy, which further compromise the benefits brought by the edge computing paradigm. To this end, we propose a measurement-based estimation solution to obtain the interference efficiently and intelligently by dynamically controlling the measurement and estimation through an accuracy-driven model. Basically, the measurement cost is reduced through the link similarity model and the channel derivation model. Compared to the exhausting measurement method, it can significantly reduce the time cost to the linear order of the network size with guaranteed accuracy through measurement scheduling and the accuracy control process, which could also balance the tradeoff between accuracy and measurement overhead. Extensive experiments based on real data traces are conducted to show the efficiency of the proposed solutions.

## 1. Introduction

Driven by the need to support ever-increasing mobile traffic and fully utilize the limited spectrum, the wireless network, especially the emerging new generation of technology, has become continuously denser to the increase in the capacity and accommodating more users. The most prominent way is either adding more cells or creating a complex structure of heterogeneous networks [1]. Apart from such the benefits brought by dense deployment and small cells, however, this results in growing inter-cell interference levels and high costs, which hinder the massive deployment of this paradigm.

Fortunately, cloud computing has been constantly evolving to provide a certain level of centralized computation and to assist the mobile network and sensor networks (mobile cloud and edge computing) [2]. Furthermore, the major portion of cloud computing facilities is owned by the ISPs and mobile communication provider, e.g., China Mobile Communication, AT&T, etc. Thus, a novel mobile network architecture Cloud Radio Access Network (Cloud-RAN) is proposed, which has the potential to answer the above-mentioned challenges faced by the mobile service provider. Cloud-RAN is seen as a typical realization of mobile network supporting soft and green technologies in the fifth generation (5G) mobile network in the year 2020 horizon [3]. In Cloud-RAN, baseband processing is centralized and shared among sites. This make it possible to adapt to non-uniform traffic and utilizes the resources’, i.e., base stations’, spectrum more efficiently. The powerful clouds behind the RAN could make previous computationally heavy resource allocation algorithms more practical and thus fully explore the potential of the capacity brought by denser deployment and small cells.

The most challenging part of this kind of method is how to model the complex interference between communications in Cloud-RAN, so that the optimization method could be leveraged to achieve good results. Two interference models are mainly proposed, which are the conflict graph model and the SINR (Signal-to-Interference plus Noise Ratio) model, respectively. No matter the conflict graph model or the SINR model, they are all based on the interference levels, which are benchmarked by RSS (Receiving Signal Strength). Most state-of-the-art work [4,5,6] mainly focused on the optimization problem on the non-convex objective function introduced by the SINR model, by assuming the RSS follows the rule-of-thumb parameter-induced signal propagation models (e.g., the path loss model [6]). However, such propagation models and the corresponding rule-of-thumb parameters cannot characterize the complex, time-varying channel conditions accurately, which in turn compromises the optimization results [7,8].

A natural question then arises: How does one measure these RSS values efficiently and accurately? An intuitive solution is using a measurement-assisted method to enhance the accuracy of the interference model for spectrum optimization.

The Cloud-RAN is featured with powerful computation capacity and massive data storage. Such an architecture is envisioned to perform a data-driven resource allocation and interference mitigation. However, to obtain accurate RSS values, exhausting measurement on all wireless links will incur an unacceptable time cost, which mainly consists of the following aspects.

- First, the optimization process in C-RAN requires the measurements of interference in every potential communication link (almost every pair of nodes) in the network, so the number of measurements grows quadratically with the network size.
- Second, due to the shadow fading and background noise, the measured interference will be dynamic over time. Therefore, it requires multiple measurements to ensure the accuracy of measurements in every link.
- Third, the signals fade differently in different channels. Thus, measurements in all channels are also required.

In summary, it takes $O(N^{2}MC)$ measurements to finish the exhausting measurement, where *N*, *M* and *C* are the network size, the number of channels and the number of measurements taken to achieve a certain accuracy, respectively.

This challenge could only be resolved with powerful centralized control and inspection capacity, which fortunately are the advantages of Cloud-RAN. By taking into account these features of Cloud-RAN, we follow the concept of “measure a few, predict many” and propose an efficient solution with accurate control for RSS estimation called the model-based solution.

Basically, the model-based solution derives the RSS values by extending the path loss model with partial measurements. It includes three steps: overhead reduction, accuracy control and measurement scheduling. The overhead reduction is based on the link similarity in propagation: with the path loss model, the Path Loss Exponent (PLE) is almost the same for a set of links in the network. Hence, only a small portion of links of such a set need to be measured, while the rest can be derived from the path loss model. Further, the measurement results of one channel can be used to compute the value of another channel. Thus, only one channel needs to be measured for each link, which reduces the measurement cost dramatically. The accuracy control is introduced for the tradeoff between the estimation accuracy and the measurement cost. The measurement scheduling is targeted at improving the time efficiency by finding the optimal schedule to assign the required measurements into different time slots and channels. With the three steps, the proposed model-based solution enables efficient interference estimation with time cost O(N), which achieves the linear order of the network size.

The contributions of this paper are summarized as follows.

- We reveal the important problem of accurate RSS estimation for data-driven resource allocation and optimization in Cloud-RAN and show the performance gap between theoretical and practical values via trace-driven experiments.
- By taking advantage of the feature of Cloud-RAN, we propose a model-based solution for efficient RSS estimation. It reduces the time cost to the level of O(N/M), where *N* is the number of nodes and *M* is the number of channels.
- We provide an accuracy control method for our solution, which achieves the required accuracy by controlling the number of measuring links. This method could help us to balance the tradeoff between the accuracy and the cost.
- We conduct extensive experiments using real communication traces collected from a wireless network testbed, which shows the efficiency of the proposed solutions.

## 2. Related Work

### 2.1. Cloud-RAN

The basic idea of Cloud-RAN was first given with the concept of the Wireless Network Cloud (WNC) by IBM [9]. It has then been further developed into today’s concept by China Mobile Research Institute [10]. According to Checko et al. [11], the major research challenges in Cloud-RAN include: high bandwidth, a strict latency network, base-station cooperation and a virtualization technique. For the first challenge, the research community mainly has focused on physical layer technology [12] and compression technology [13]. The community has also devoted research effort toward developing high-end base stations and related technology to synchronize them [14]. Compared to the two previous research challenges, virtualization technology, which is indispensable in realizing Cloud-RAN, has attracted the most research efforts. In Sigcomm 2013, Yang et al. [15] proposed a soft-defined RAN architecture via virtualization. The researchers from Stanford University also proposed SoftRAN [16] to apply the SDN and virtualization technology. Recently, Zhou et al. [17] proposed a hyper-cellular architecture for Cloud-RAN-like applications.

### 2.2. Wireless Network Optimization

The SINR model has been applied widely to characterize the interference for resource allocation in wireless networks. Although regarded as a better model, there are still many drawbacks in carrying out optimization with such a model due to the computational complexity it introduces. As a result, many state-of-the-art efforts on network optimization were based on single-hop networks, e.g., [4,18]. For multi-hop networks, most of the existing studies focused on cross-layer optimization problems. For example, in [5], Bhatia and Kodialam optimized power control and routing under the assumption that some frequency hopping mechanisms are in place for scheduling, which simplified scheduling hardness. For cross-layer optimization based on the SINR model, almost all existing efforts (e.g., [19,20,21]) followed the layer-decoupled approach for analysis. With this approach, the solution was obtained by determining an algorithm/mechanism for one layer at a time and then piecing them together without the need of solving a joint optimization problem. Due to decoupling in the solution procedure, these approaches are heuristic and cannot offer performance guarantee.

### 2.3. RSS Estimation

In [22], the authors applied the dynamic programming concept with storing the path loss for future use. They proposed an interpolation algorithm to estimate the path loss between sensors. In [23], the authors proposed a PLE estimator-based algorithm to facilitate a fast handover algorithm. Based on these works, probability scheduling based on a distance estimator was discussed in [24,25], which assumed that the distance between two neighboring nodes is known. Our previous work [7] focused on using the path loss model to improve SINR-based optimization in wireless networks, where the path loss model is using the rule-of-thumb parameters.

All the aforementioned works endeavored to craft interference models that simplify the problem complexity. However, none of them focused on making a swarm measurement on all potential links and all channels, which could lead to a better solution for the throughput optimization in wireless networks. Our work is inspired by these research works and work on the topic of the interference measurements and estimation to assist the resource allocation and optimizations in Cloud-RAN.

## 3. Problem Formulation

We consider a synchronized, time-slotted wireless network consisting of *N* nodes denoted by N. A set of channels, denoted by M, is available for each node in N. We denote *P* as the transmitting power of each node operating over any channel and $p_{ij}^{m}$ as the RSS of a signal from node i∈N over channel m∈M received at node j∈N.

Our main task is to obtain all the RSS values over each pair of nodes and each channel, i.e.,

(1) $$\{p_{ij}^{m}|i,j\in N,i\neq j,m\in M\}.$$

With such information, we can calculate all the RSS values over any link (i,j)∈N×N and any channel m∈M under any arbitrary channel assignment configuration. More precisely, let $N_{m}$ be the set of nodes operating over channel *m*. Then, the SINR value over link (i,j) and channel *m*, denoted by ${\mathrm{SINR}}_{ij}^{m}$, could be determined by:

(2) $${\mathrm{SINR}}_{ij}^{m}=\left\{\begin{array}{cc} \frac{p_{ij}^{m}}{N_{0}+\sum_{k\in N_{m},k\neq i}p_{kj}^{m}} & \mathrm{if}\;i\in N_{m}\\ 0 & \mathrm{otherwise}, \end{array}\right.$$

where $N_{0}$ is the ambient normalized Gaussian noise density, and it can be further used to determine the network performance (e.g., aggregate throughput, fairness, etc.) via the well-known Shannon capacity formula [26,27]. Therefore, the network performance could be optimized by choosing a proper channel assignment configuration.

Our goal is to measure the $p_{ij}$ with a certain accuracy. However, how to benchmark such accuracy still needs further study. In our paper, we mainly benchmark our measurement scheme via the following metrics:

- Time cost: the number of time slots *t* to finish all the measurements;
- Measurement overhead: the quantity of the measurements conducted. Note that the measurements could be performed simultaneously, and the measurement overhead should be larger or equal to the time cost;
- Accuracy: the accuracy metric in our algorithm could be divided into link-wise accuracy and network-wide accuracy, respectively. The link-wise accuracy implies that the measurements $\left\{p_{ij}^{m}\right\}$ should be controlled within the confidence 1−α/2. The network-wide accuracy implies that the β portion of the measurements is accurate with a certain confidence.

The accuracy is ensured by an adequate number of measurements, while the overhead and time cost metric require as few measurement as possible. Thus, our target is to design the solution that achieves good tradeoffs among these metrics.

## 4. Solution

In this section, we introduce our solution, which could measure the RSS values efficiently and accurately. As previously mentioned, our basic idea is to follow the concept of “measure a few, predict many”. This idea is mainly enabled by exploiting the inherent correlations between the RSS values of different links and channels to reduce the measurement overhead. Our solution is performed both on the base-station side and the cloud side. The model-based solution mainly consists of the following steps:

- Firstly, we reduce the total measurement overhead by reducing the number of links and channels that conduct measurements.
- Secondly, we derive the relationship between the number of measurements and the accuracy we could achieve. The accuracy control could help us to balance the tradeoff between the accuracy and measurement overhead.
- Then, based on the results of the previous two steps, we manage to reduce the time cost by distributing the non-conflict measurements into time slots and channels.

With these three steps, we manage to achieve the targets that obtain the SINR in all potential links and channels accurately and efficiently. We now present our solution in detail for all three steps.

### 4.1. Overhead Reduction

We manage to achieve low measurement overhead in two dimensions:

- Measurements in links: First, we reveal that a set of links in the network shares the same (or very close) propagation property via the path loss model, e.g., close path loss exponent. Thus, we only need to perform the measurement over small portion of links for this set. For the whole network, we could select a small portion of links called representative links for measurement, thus reducing the measurement overhead.
- Measurements in channels: Second, the measurement overhead could be further degraded by only measuring a single channel in each link, as the propagation characteristics in other channels could be derived from the measured channel.

#### 4.1.1. Reduction in Measurement of Links

As previously mentioned, the basic idea behind this is that several coexisting links may share the same propagation property. Now, we show the correlation of propagation properties between different links. This is modeled with the Log-distance path loss model, which is defined as [28]:

(3) $$p_{ij}^{m}=P+PL_{0}+10\gamma \log d_{ij},$$

where $d_{ij}$ is the distance between node *i* and node *j*; Pis the transmitting power of a wireless device; and γ is the Path Loss Exponent (PLE). $PL_{0}$ is the path loss in the reference distance. We apply the path loss model here without any a priori knowledge of γ, but determine it through measurement. Thus, the measurements of $p_{ij}^{m}$ could be transformed into estimating the γ. Note that, in this solution, we have an additional assumption of a priori knowledge of nodes’ positions.

Generally speaking, certain links may share the same or similar γ. Thus, we can group all the links in the network into multiple clusters and pick two or three links from every cluster as representative links. The clustering method is given below.

1. Take a small number of measurements in the form of the sequential broadcast of the measurement packet in each node. This number of measurements should be much smaller than the number required to achieve the accuracy requirement, as given in the next subsection;
2. Estimate the PLE γ for each link via linear regression;
3. Apply the *k*-means algorithm to cluster these links into *k* groups by using the PLEs as the metric, where *k* is the number of representative links.
4. Select the representative links for each group.

Here, *k* is a tunable parameter for balancing the measurement overhead and accuracy. Once the representative links have been identified, they will be measured in a more accurate manner via the accuracy control mechanism shown in the next subsection. Note that we choose more than one representative link for each set, and this will result in more accurate estimation of γ for this set.

To make a better presentation of the path loss model, we introduce a coefficient θ to replace $P+PL_{0}$ and rewrite Equation (3) as:

(4) $$p_{ij}=10\gamma \log d_{ij}+\theta .$$

Equation (4) shows that the RSS value is a linear function of the Log-distance between a pair of nodes. For a set of links in the same cluster, they have the same coefficients γ and θ. Thus, if the coefficients are known, we can make a good estimation of RSS values of all links in a cluster. Note that there are two coefficients in the model. Then, we must pick more than two representative links for one cluster.

To derive γ and θ, we can simply choose a few representative links in the cluster and make several measurements on such links (the number of measurements is decided by the accuracy control method introduced in a later section). With the measured value, we can fit them into Equation (4) and use linear regression to obtain the values of γ and θ. Therefore, the RSS values of the other unmeasured links in the same cluster can be estimated using Equation (4) efficiently.

#### 4.1.2. Reduction of the Measurements in Channels

Now, we introduce how to reduce the number of channels with which to conduct the measurements. This is mainly enabled by introducing a model that drives the RSS in the channels from the measurements in other channels over the same link. This model is a frequency-related signal propagation model [28] and is formalized as:

(5) $$p_{ij}^{m}=10\log_{10}f_{m}^{2}+10\gamma \log_{10}d_{ij}-\phi_{0}\left(in\;dB\right),$$

where $f_{m}$ is the frequency of channel *m* and $\phi_{0}$ is the additional loss, which is constant.

Suppose $p_{f_{1}}$ is the RSS at $n_{2}$ when the transmission uses the carrier frequency $f_{1}$, while $p_{f_{2}}$ uses $f_{2}$. With (5), we can obtain the following equation:

(6) $$p_{f_{1}}-p_{f_{2}}=20\log\left(\frac{f_{2}}{f_{1}}\right).$$

Suppose that $f_{1}<f_{2}$, then with this equation, either $p_{f_{1}}$ or $p_{f_{2}}$ is known, and the other can be inferred.

### 4.2. Accuracy Model and Control Mechanism

In this subsection, we introduce how to achieve link-wise accuracy by controlling the number of samples in each selected channel of representative links.

Generally, we take several samples in the representative links, then we use the average of the samples as the value of the RSS in the corresponding link. Given several of representative links, we can control the overall accuracy in the following steps. Firstly, we derive the required number of samples $m_{ij}$ for the measurement accuracy corresponding to the representative link (i,j). Then, we compute the sample size $n_{ij}$ that is required to ensure the estimation accuracy of the link pairs that are clustered with (i,j). Finally, we decide the overall sample size by max{$m_{ij}$, $n_{ij}$}, which is elaborated to ensure the required measurement accuracy and estimation accuracy.

Regarding the measurement accuracy, we have the following lemma.

**Lemma** **1.** *To ensure that the accuracy of the measured value ${\bar{p}}_{ij}$ is within (1−β) of the true value in the confidence level of (1−α), the minimum required samples $m_{ij}$ for the measurement accuracy corresponding to the representative link (i,j) are $\frac{z_{1-\alpha /2}^{2}\sigma^{2}}{{\bar{p}}_{ij}^{2}\beta^{2}}$*.

**Proof.**  Suppose that measurement error is within the β portion of the mean at the 1−α confidence level for one link (i,j) and the mean value of the measured RSS is ${\bar{p}}_{ij}$. Then, with a set of $m_{ij}$ samples on link (i,j), the 1−α confidence interval for the mean RSS could be derived with:

(7) $${\bar{p}}_{ij}\mp z_{1-\alpha /2}\frac{\sigma }{\sqrt{m_{ij}}},$$

where $z_{1-\alpha /2}$ is the (1−α/2)-quantile of the standard normal variate and σ is the standard deviation of samples. To satisfy the required accuracy, it requires:

(8) $$z_{1-\alpha /2}\frac{\sigma }{\sqrt{m_{ij}}}\leq {\bar{p}}_{ij}\beta .$$

Solving this inequality, we have:

(9) $$m_{ij}\geq \frac{z_{1-\alpha /2}^{2}\sigma^{2}}{{\bar{p}}_{ij}^{2}\beta^{2}}.$$

Therefore, $\frac{z_{1-\alpha /2}^{2}\sigma^{2}}{{\bar{p}}_{ij}^{2}\beta^{2}}$ samples of packet transmission status are enough to ensure the required accuracy of (1−β) for the link (i,j). ☐

Note that, here, σ and ${\bar{p}}_{ij}$ can be calculated from the samples collected in the process of determining the representative links.

Regarding the estimation accuracy, the minimum required $n_{ij}$ is obtained as follows. Let C denote the cluster containing the representative (i,j). To ensure the accuracy of the estimated RSS value $p_{lh}^{\prime }$, which is calculated with the linear regression of Equation (4), for other links (l,h)∈C with an accuracy of (1−φ) at the confidence level of (1−α), we have the following lemma.

**Lemma** **2.** *To ensure that the estimated RSS value $p_{lh}^{\prime }$, with an accuracy of (1−φ) at the confidence level of (1−α), the minimum required sample size in link (i,j) is:*

(10) $$\lceil \frac{s_{e}^{2}z_{1-\alpha /2}^{2}}{p_{lh}^{\prime 2}\varphi^{2}}\left[1+\frac{{\left(\log d_{lh}-\langle \log d\rangle \right)}^{2}}{\langle \log^{2}d\rangle -{\langle \log d\rangle }^{2}}\right]\rceil .$$

*where $\langle \log d\rangle =\frac{\sum_{i=0}^{n_{C}}\log d_{i}}{n_{C}}$. Here, $d_{i}$ denotes the length of the representative link where $p_{i}$ was measured. The notation $d_{lh}$ stands for the length of link (l,h) and $s_{e}=\sqrt{\frac{\sum_{i=1}^{n_{C}}{\left(p_{i}-10\gamma \log d_{ij}-\theta \right)}^{2}}{n_{C}-2}}$*.

**Proof.**  Given $n_{C}$ measurements in total $\left\{p_{i};i=1\ldots n_{C}\right\}$ on the representative links of C, we can drive the PLE γ for C with linear regression on the model in Equation (4). Because there are two regression parameters, the degree of freedom is $n_{C}-2$. Hence, the corresponding standard deviation of errors for this linear model is $s_{e}=\sqrt{\frac{\sum_{i=1}^{n_{C}}{\left(p_{i}-10\gamma \log d_{ij}-\theta \right)}^{2}}{n_{C}-2}}$.

For each unmeasured link (l,h)∈C, the estimated RSS $p_{lh}^{\prime }$ could be derived with Equation (4). According to [29] (Section 14.6), the standard deviation of $p_{lh}^{\prime }$ is:

(11) $$s_{p_{lh}^{\prime }}=s_{e}{\left[\frac{1}{n_{C}}+\frac{{\left(\log d_{lh}-\langle \log d\rangle \right)}^{2}}{\sum_{i=0}^{n_{C}}\log^{2}d_{i}-n_{C}{\langle \log d\rangle }^{2}}\right]}^{1/2},$$

where $\langle \log d\rangle =\frac{\sum_{i=0}^{n_{C}}\log d_{i}}{n_{C}}$. Here, $d_{i}$ denotes the length of the representative link where $p_{i}$ was measured and $d_{lh}$ stands for the length of link (l,h). In our implementation, we set the same sample size for all the representative links of C. Hence, no matter how large $n_{C}$ is, the 〈logd〉 is always equal to the the average log-length of the representative links of C. Then, 〈logd〉 can be determined without knowledge of $n_{C}$. Similarly, we can transform $\sum_{i=0}^{n_{C}}\log^{2}d_{i}$ into $n_{C}\langle \log^{2}d\rangle$. Then, we transform Equation (11) into:

(12) $$s_{p_{lh}^{\prime }}=\frac{s_{e}}{\sqrt{n_{C}}}{\left[1+\frac{{\left(\log d_{lh}-\langle \log d\rangle \right)}^{2}}{\langle \log^{2}d\rangle -{\langle \log d\rangle }^{2}}\right]}^{1/2}.$$

Regarding the accuracy constraint, we have the 1−α confidence level for $p_{lh}^{\prime }$ is $p_{lh}^{\prime }\mp s_{p_{lh}^{\prime }}t_{\left[1-\alpha /2,n_{C}-2\right]}$. Here, $t_{\left[1-\alpha /2,n_{C}-2\right]}$ is the (1−α/2)-quantile of a t-variate with $n_{C}-2$ degrees of freedom. Assuming that the estimation error requirement is within φ of the mean value at the 1−α confidence level, the following inequality will hold:

(13) $$s_{p_{lh}^{\prime }}t_{\left[1-\alpha /2,n_{C}-2\right]}\leq p_{lh}^{\prime }\varphi .$$

Combing (12) and (13), we have the following result on $n_{C}$:

(14) $$n_{C}\geq \frac{s_{e}^{2}t_{\left[1-\alpha /2,n_{C}-2\right]}^{2}}{p_{lh}^{\prime 2}\varphi^{2}}\left[1+\frac{{\left(\log d_{lh}-\langle \log d\rangle \right)}^{2}}{\langle \log^{2}d\rangle -{\langle \log d\rangle }^{2}}\right].$$

Let $n_{d_{lh}}$ equal the right part of (14), which varies with different $d_{lh}$. Thus, the required sample size $n_{C}$ for C should be larger than $\max_{(l,h)\in C}n_{d_{lh}}$. Note that in $n_{d_{lh}}$, the t-variate is still related to $n_{C}$. To eliminate this, we estimate $t_{\left[1-\alpha /2,n_{C}-2\right]}$ by $z_{1-\alpha /2}$, since $t_{\left[1-\alpha /2,n_{C}-2\right]}$ is very close to $z_{1-\alpha /2}$ for larger $n_{C}$ (e.g., $n_{C}\geq 20$). Then, we set the sample size for each representative link (i,j) as the average of total required measurements, $n_{ij}=\left\lceil n_{C}/b\right\rceil$, where *b* is the number of representative links for C. ☐

With Lemmas 1 and 2, we can easily derive the following theorem.

**Theorem** **1.** *The required sample size to guarantee the accuracy of both measured and estimated RSS within (1−β) and (1−φ) at the confidence level of (1−α) for (i,j) is $\max\{n_{ij},m_{ij}\}$*.

Theorem 1 endows us with the ability to control the accuracy with minimum costs. The accuracy control process should be conducted before the measurement.

### 4.3. Time Efficiency

The time efficiency is achieved through measurement scheduling, which arranges the measurement into different slots and channels to perform non-conflicting simultaneous measurements in the least time slots. This conflict-free scheduling can achieve time efficiency and in the meantime can help to identify the signal source when the SNR is too small to decode the signal.

Since different sampling sizes are derived for different representative links, we need to take this into account for measurement scheduling. We use a matrix $\left\{c_{ij}\right\}$ ($c_{ij}\in I$) to represent the calculated sample size in link (i,j), and $c_{ij}=0$ if (i,j) is not selected as the representative link.

We now formally define this problem as:

**Definition** **1.** *Minimum time, accuracy constraint measurement problem: Given a network N with N nodes and M channels M, find the schedule with earliest end time T subject to the measuring requirement defined in $\left\{c_{ij}\right\}$*.

Assume $x_{i,t}^{m}\in \left\{0,1\right\}$ is a binary indicator, where $x_{i,t}^{m}=1$ denotes that node *i* performs a broadcast in channel *m* at time slot *t*. The problem can be formulated as:

(15) $$\begin{array}{cc} \mathrm{Min}\;T & \\ s.t. & \sum_{m\in M,t\leq T}\left(x_{i,t}^{m}-x_{i,t}^{m}x_{j,t}^{m}\right)\geq c_{ij},\forall i,j\in N \end{array}$$

(16) $$\begin{array}{c} \sum_{m\in M}x_{i,t}^{m}=1,\forall i\in N,t\leq T \end{array}$$

Here, the successful measurement is when node *i* is measuring in channel *m* while other nodes, say *j*, do not. Thus, Equation (15) implies that the cost of the total measurements is equal to the calculated sample size in link (i,j). Equation (16) implies that we only need to select one channel in one link to perform the measurement; while the others could be derived by the cross channel measurement method in Section 4.1.

However, the problem formulated above is an integer non-convex optimization problem with n uncertain number of variables. Generally, it is NP-hard and unable to obtain the result in polynomial time. To get the approximate result efficiently, we divide the scheduling process into two steps.

- First, since only representative links are obliged to be measured, we derive the number of broadcasting nodes to cover all the representative links in the node-wise schedule.
- Second, we study how to distribute the measurements into different channels, such that the whole measurement process will end in the earliest time.

Regarding how many nodes are enough to cover all the representative links, we mainly transform the problem into a minimum dominating set problem. Let $L_{r}$ denote the set of representative links. We construct a graph $G_{L_{r}}$ with nodes being the union of nodes whose links are in $L_{r}$, and the edges are the links in $L_{r}$. Hence, trivially, the minimum required nodes to cover all the representative links are the minimum dominating set of $G_{L_{r}}$. This is a classic NP-hard problem [30]. However, there are many fast approximation algorithms proposed. Here, we adopt the algorithm in [31], which achieves constant-time complexity. The output node set is denoted as $N_{r}$.

**Figure 1:**
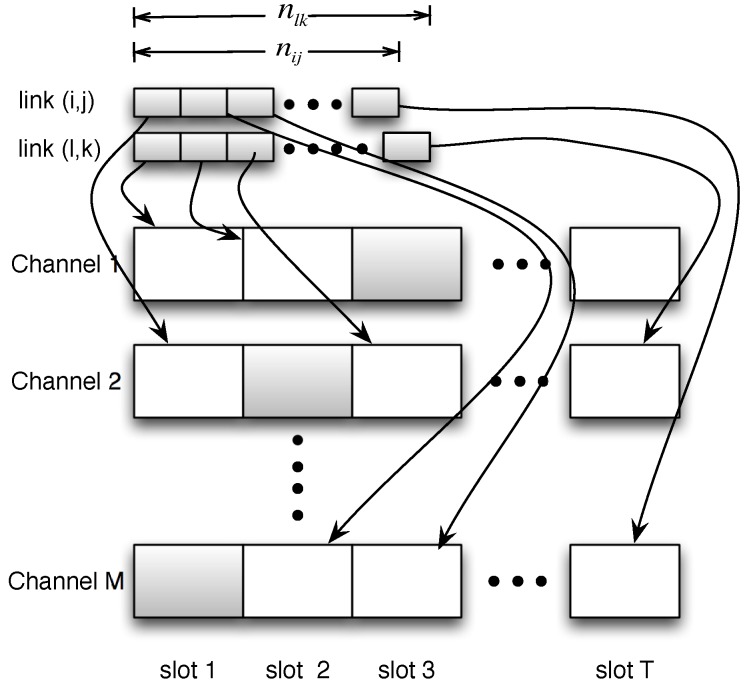
Illustration of the balanced partition modeling for measurement scheduling.

Then, with $N_{r}$, we study how to distribute their corresponding measurements into *M* channels to achieve time efficiency. We prove it to be a balanced partition problem [30]. The problem can be formally defined as follows. Given $|N_{r}|$ nodes and their corresponding sample size $\left\{c_{i}|i\in N_{r},c_{i}=\max_{j\in N}c_{ij}\right\}$, we want to divide $\left\{c_{1},c_{2},\ldots ,c_{|N_{r}|}\right\}$ into *M* sets, say $\left\{S_{1},S_{2},\ldots ,S_{M}\right\}$, such that $\max_{i\leq M}∥S_{i}{∥}_{l_{1}}$ is minimized. Here, $∥S_{i}{∥}_{l_{1}}$ is the sum of all elements in $S_{i}$. In this formulation, this problem is precisely a balanced partition problem, which is extensively studied and proven to be NP-complete [30]. This kind of partition modeling of the measurement scheduling problem is illustrated in Figure 1. The partition problem is often referred to as “the easiest NP-complete problem” [30]. Many efficient approximate solutions have been proposed. We apply the recent proposed heuristic algorithm: LRM [32], which achieves O(NlogN) time complexity and produces better results in the scenario of large node size.

With the above partition result, we can generate the measurement schedule in the following way: we first determine the channel of measurement for the nodes in $N_{r}$ according to their group number *m*. The time sequence of measurement in one channel could be random. Then, we set $x_{i,t}^{m}=1$ if node *i* is scheduled to broadcast with channel *m* in time slot *t*.

We now derive the time efficiency we could achieve, which is summarized as the following theorem.

**Theorem** **2.** *The measurement scheduling algorithms described above could ensure the time efficiency at the level of O(N), and this is upper-bounded by $\left\lceil \frac{|N_{r}|C}{M}\right\rceil$*.

**Proof.**  Since there are $|N_{r}|$ broadcasting nodes and each node needs to take $c_{i}\;\left(i=1,\cdots ,\right|N_{r}\left|\right)$ samples, the total number of required measurements is $\sum_{i=1}^{|N_{r}|}c_{i}$. The measurements can be distributed in *M* channels simultaneously, so the measurements can be finished in $\frac{\sum_{i=1}^{|N_{r}|}c_{i}}{M}$ time slots with our scheduling algorithm. Let $C=max\{c_{1},c_{2},\cdots ,c_{|N_{r}|}\}$, and the above number of time slots is upper-bounded by $\left\lceil \frac{|N_{r}|C}{M}\right\rceil$. In most wireless networks, the number of channels *M* is a fixed constant. The sample size *C* is a controllable value, which is independent of the network size, and can be considered as a constant once the required accuracy is given. Thus the time efficiency is $O(|N_{r}|)$. Since $N_{r}$ is a subset chosen from N, we have $|N_{r}|<N$. In conclusion, the time efficiency of the proposed algorithm is O(N), which is linear to the network size. ☐

## 5. Evaluation

In this section, we analyze the performance of the proposed solutions via experiments. We first present the experimental methodology and simulation settings; then, we discuss the numerical results.

### 5.1. Experiment Targets

We designed the experiments to examine the modeling-based efficient RSS measurement in the following aspects:

- The primary target is to evaluate how our solutions impact the SINR-based throughput optimization algorithms.
- We also want to quantify the measurement overhead and time cost.
- The link-wise accuracy should be examined.

### 5.2. Experimental Settings

Our experiments’ methodology is basically a real dataset-driven simulation. Thus, in this section, we mainly introduce the detail of the datasets, the processing of the data and the simulation settings.

We pick two representative dataset for the outdoor scenario and the indoor scenario.

- SWIMdataset: The first one is our data collection from the SWIM platform [33], which mainly consists of 10 wireless nodes running in 802.11a/b/g mode. We collected the RSSI of the broadcasting beacons from each AP. Each node will be activated to broadcast the beacon and tuned to 11 different channels sequentially. Then, a laptop will move to 25 different locations (including the locations of 10 APs) and collect more than 50 beacons in two minutes from one AP in each channel. We also collect the AP ID and channel ID at the same time. This dataset is a representative indoor dataset, while the redundant collected data are very useful to mitigate the interference from the other WiFi access point in the building. A more detailed floor plan and the deployment can be found in [33].
- MetroFidataset: The other is the MetroFi dataset [34], which covers 30,991 measurement locations from 70 APs with known locations and generates more than 200,000 samples. This dataset is basically an outdoor collected dataset. This dataset is collected from a municipal wireless mesh network in Portland, Oregon. The deployers collect signal strength measurements using a battery-powered embedded computer with an external 7-dBi omnidirectional antenna and a GPS device. The collector roams around network to different locations. This dataset can be found in the crawdad database.

In order to simulate the Cloud-RAN scenario to examine the effectiveness, we use the real collected data to generate several experimental scenarios from these datasets. Note that the SWIM dataset is from an indoor deployment, and MetroFi is from an outdoor deployment. Thus, we combine both datasets to generate our experimental scenario. The experimental scenarios consist of 5, 10, 15 and 20 different nodes. The operating spectrum is 2.4 GHz, with 11 channels of a bandwidth of 20 MHz. The locations are mapped into a 2-km square area, with several indoor and outdoor deployed APs. The RSS between the indoor and the outdoor APs are computed from the model in [28] with the parameters computed from both scenarios. More than 200 scenarios were generated to perform a statistical performance evaluation. In addition, the throughput of the Cloud-RAN network in the given area is computed with the algorithm in [27].

The basic benchmark to evaluate our and the comparison solution is the optimized throughput using the estimated RSS values. The accuracy is quantified using the MPE (Mean Percentage Error), which is formally defined as: $\frac{1}{N}\left(\frac{p_{ij}^{\prime }-p_{ij}}{p_{ij}}\right)$. Here, $p_{ij}^{\prime }$ is the estimated RSS for (i,j).

We also evaluated the parameter performance. There are mainly two parameters in our solution. The first one is *k*, standing for the number of representative links. According to our solution, it has a linear relation with the total links. Aiming at a fair comparison for the solution applied to different scenarios with different link numbers, this parameter is altered to $k^{\prime }=k/N$. The second parameter is *u*, denoting the number of channels selected to perform measurement in every representative link. Finally, regarding the accuracy requirement, in our experiments, it is uniformly set to a 5% error rate within a 95% confidence level.

All the simulations and data processing were conducted in MATLAB 2012b on a server equipped with a CPU of Intel i7 4790 and 16 GB DDR3 RAM. As the channel condition and the network optimization in our algorithm were protocol independent, we choose to examine the algorithm performance rather than the protocol level performance. Consequently, we used MATLAB to simulate the experiments. This included the distributed measurement collection, measurement scheduling and the result of the estimated RSS, upon which the throughput optimization algorithm was conducted.

### 5.3. Experiments Results

#### 5.3.1. Performance of Overall Solution

**Figure 2:**
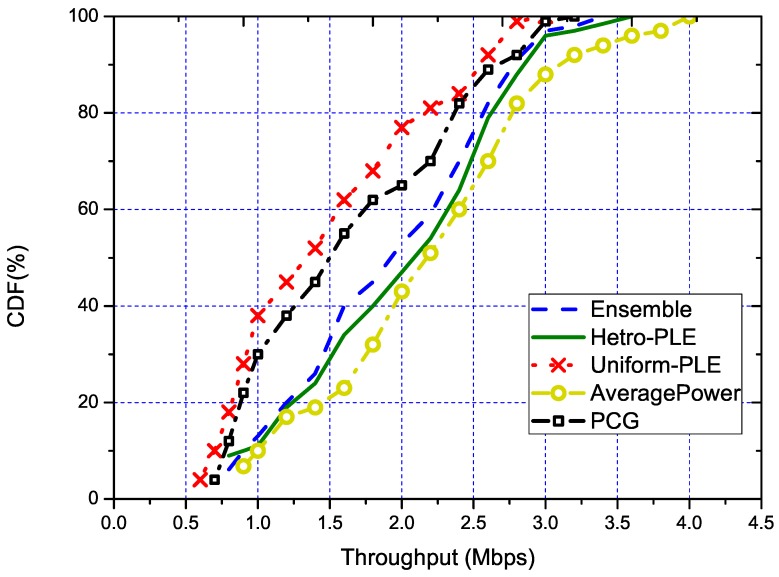
The CDF of throughput using different RSS estimation algorithms. PLE, Path Loss Exponent.

We evaluate the performance of our solution in two dimensions. The first dimension is the performance improvement of the throughput optimization algorithms brought by the estimated RSS from our solution. This improvement is mainly quantified by the algorithm in [27] under different RSS estimation methods on all the generated scenarios. The comparison result is shown in Figure 2. In this graph, the line “AveragePower” is the result using the RSS of averaging the collected real-power data without estimation. Thus, this line could serve as the baseline result. The line named “Uniform-PLE” is the throughput under the RSS estimated with merely the path loss model with the rule-of-the-thumb PLE value throughout the network. Then, the line named “Hetro-PLE” is the one with the path-loss model using a distinct PLE parameter from link to link. Because this type of setting will lead to a larger variable space but cost much more than the Uniform-PLE, this line could serve as the optimal result achieved by the path loss model. The result of our solution is the line named “Ensemble”, which is performed under the parameter of $k^{\prime }=1/4,\;u=1$. Overall, our solution is very close to the optimal result “Hetro-PLE”. Specifically, the numerical results show that our solution could achieve 94% of that under “Hetro-PLE” on average. In addition, we can also see from the figure that our solution performed much better than the traditional path loss model using the rule-of-the-thumb parameter.

In fact, to improve the throughput of the wireless network or RAN, another set of representative works is on the conflict graph [8,35]. The conflict graph-based solution features fast resolving time. However, our solution is optimization algorithm independent. Thus, we only examine the effects on how the solution impacts the optimization results. We choose the solution in [8], where the practical conflict graph is proposed. The PCG(Practical Conflict Graph)features very flexible signal strength estimation and uses only the conflict graph to encounter the accumulative interference effect. To make an even comparison to our solution, we conduct the PCG with the graph coloring algorithm in the same set of scenarios of our former experiment. The results are also shown in Figure 2, from which we can see that the line “PCG” is just below the line of “Uniform-PLE”. This result shows that the “PCG” indeed provides a better result in estimation of the interference in the network. This result is also a little bit of surprise, as the PCG only represents the conflict in binary, which is a coarse result. However, the optimization result is better than the rule-of-the-thumb driven model-based optimization. This result provides an insight of how much the optimization results rely on the accuracy of the interference estimation. We can also see that our solution is much better than PCG. This is mainly due to our solution being a much finer-grained estimation of the RSS with larger representation accuracy. Secondly, the coloring algorithm in PCG is only an approximate algorithm, which also compromises the results in PCG.

**Figure 3:**
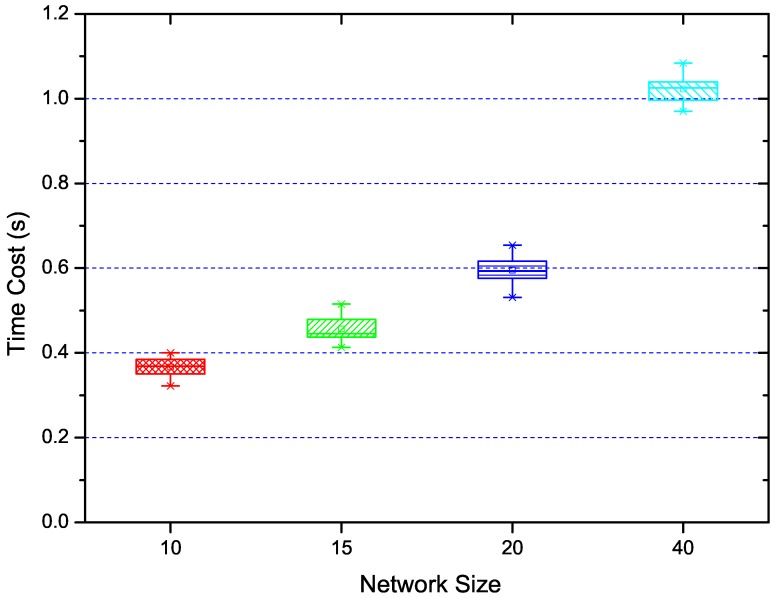
The time cost to conduct our algorithms in scenarios with different network sizes.

We also evaluate the time cost of our solution in different scenarios. Specifically, we conduct our algorithm, including representative link selection, accuracy control, measurement scheduling in more than 120 scenarios, with 30 each for network sizes of 10, 15, 20 and 40 nodes. All the algorithms are conducted sequentially in different scenarios, and we use the time counter in MATLAB to get the time costs. The parameter setting of this experiment is that k’ = 1/4 and u = 3. The results are displayed in Figure 3. From this figure, we can see that the time cost grows with the network size. This is mainly due to two reasons. Firstly, the representative links grow linearly with the network size. Secondly, the measurement scheduling is conducted in the cluster with different representative links. The most time cost is contributed by the measurement scheduling algorithm.

**Figure 4:**
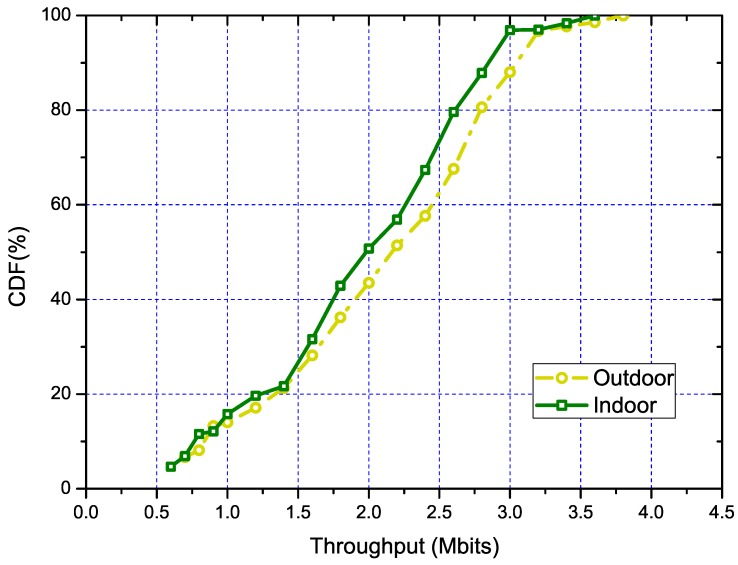
The CDF of throughput under different scenarios for indoors (SWIM) and outdoors (MetroFi).

Regarding the performance of our algorithm in outdoor and indoor scenarios separately, we conducted statistical experiments. The experiments were conducted in 30 scenarios each for the SWIM dataset and MetroFi dataset. Same as the overall experiments, we used the throughput optimized result as the benchmark to quantize the performance of our algorithm. The results are shown in Figure 4, from which we can see that in the smaller sized network, our algorithm performed almost the same between indoor and outdoor scenarios. Meanwhile, when the network size grew (indicated by the increase of network throughput), our algorithm performed much better in the scenario of the outdoor environment. This is mainly due to that the core of our solution being the PLE-based model, which is more accurate in outdoor environments [28]. Moreover, in the indoor environment, the signal propagation is much more complex. The signal propagation model cannot capture the interference from the unknown signal source, which is much denser in the indoor environment.

#### 5.3.2. Performance of the Overhead Reduction

**Figure 5:**
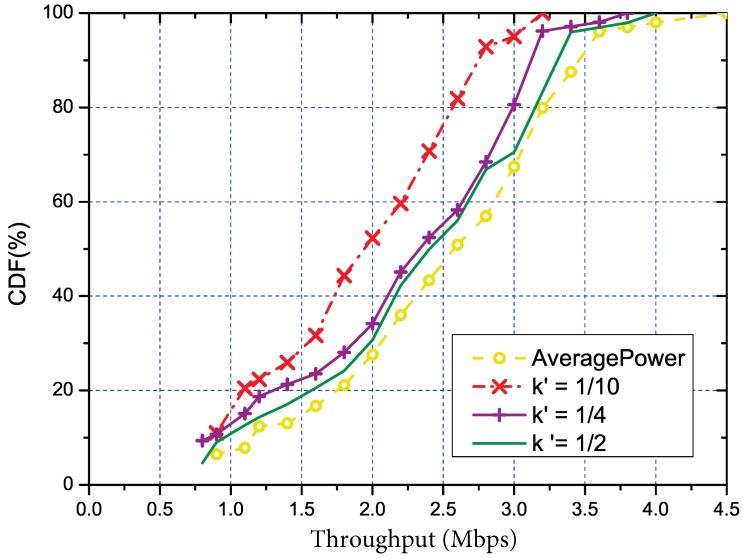
The CDF of throughput under different $k^{\prime }$, where $k^{\prime }$ stands for the ratio of representative links to the total number of links.

**Figure 6:**
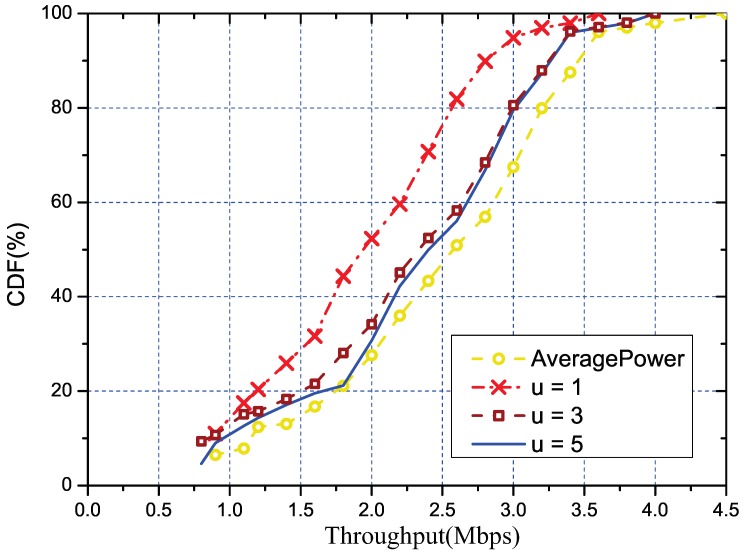
The CDF of throughput under different *u*, where *u* denotes the number of channels selected to perform the measurement in every representative link.

**Figure 7:**
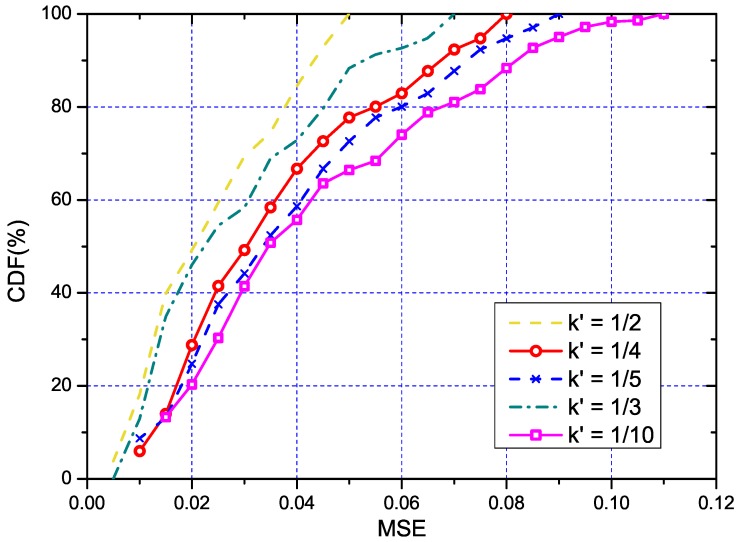
The CDF of estimated MPE under different numbers of representative links.

As previously mentioned, we also studied how the parameters $k^{\prime }$ and *u* affected our solution and tried to find the proper settings for them. Same as the the overall evaluation, we also used the throughput to quantify the parameters, and the results are respectively shown in Figure 5 (with u=1) and Figure 6 (with $k^{\prime }=1/5$). Trivially, both parameters are linearly related to the measurement cost. The estimation MSE (Minimum Square Error) under different $k^{\prime }$ is shown in Figure 7. We used the MSE to quantify the error mainly due to the regression method being used to estimate the RSS of the other links. From the figure, we can tell that a small number of $k^{\prime }=1/4$ is enough to control the error to an acceptable degree. In addition, we can also see that with the increasing of $k^{\prime }$ and *u*, the performance of our solution would also increase. Meanwhile, the link with $k^{\prime }=1/4$ and u=1 was close enough to the optimal solution. Such settings could be regarded as reference configuration parameters for our solution.

#### 5.3.3. Performance of Accuracy Control

**Figure 8:**
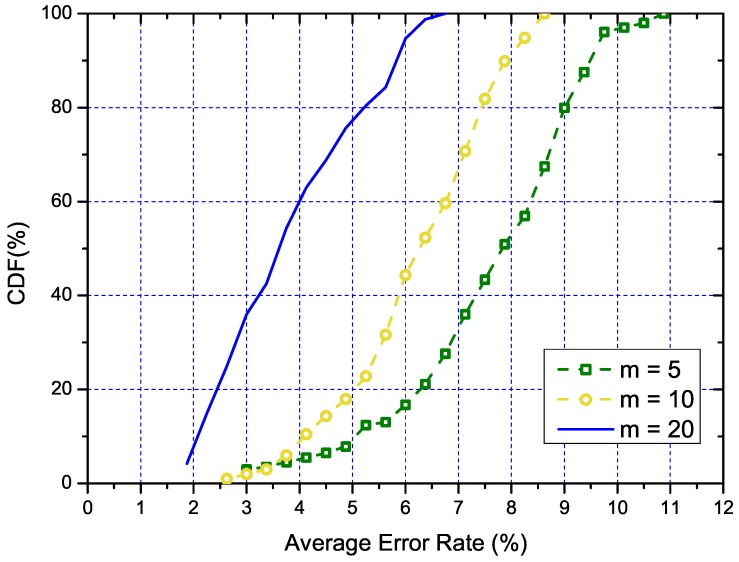
The CDF of the estimated error rate for different numbers of measurement.

As part of our solution and the main contribution, we also examine the performance of the accuracy control. Figure 8 illustrates the CDF of the average measurement error. We can tell from the figure that the cost to control the error to an acceptable level is small. For instance, to control the average error to less than 9% only requires a sample size of 10. Furthermore, we can also tell from the figure that with the increase of the number of samples collected, the measurement error further decreases. Specifically, with only 20 samples, the error rate is usually smaller than 5%. In summary, the proposed accuracy control method can save significant sampling overhead since it only takes a very small number of measurements in general.

#### 5.3.4. Performance of Measurement Scheduling

**Figure 9:**
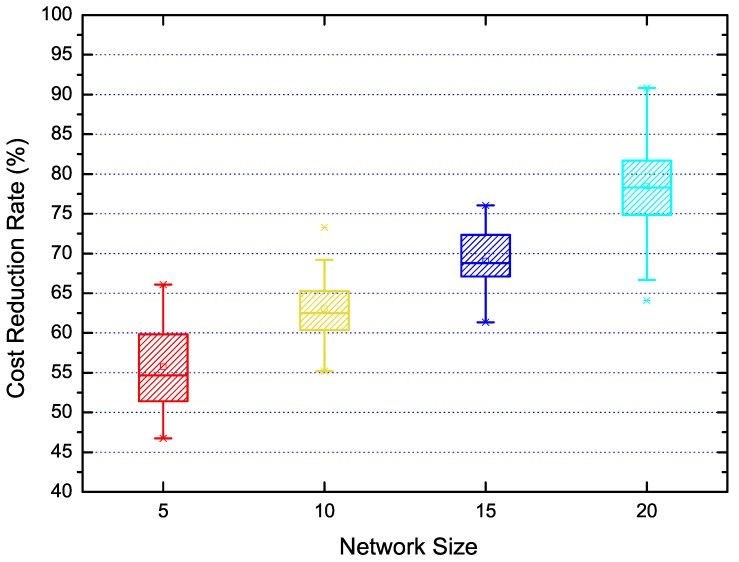
The cost reduction by measurement scheduling under different network sizes.

The proposed measurement scheduling algorithm in our solution was designed to reduce the time cost. We examined this algorithm in scenarios of 5, 10, 15 and 20 nodes respectively and performed statistical results analysis, which are shown in Figure 9. Here, the Y-axis is the normalized time cost reduction ratio. This figure mainly illustrates that the rate of cost reduction scales with the network size. Specifically, the measurement time cost could decrease as much as 90% when the network size is 20 nodes.

From the above analysis, we conclude that the our solution is effective and could significantly reduce the time cost in a controllable manner.

## 6. Conclusions

Cloud-RAN is envisioned to solve the challenges arising from the increasing transmission demand of wireless communication networks in a cost-effective way. It also has the potential to perform a data-driven resource allocation among cells such that the communication capacity could be maximized with controlled inter-cell interference, which is a major issue in today’s denser deployment. However, such a data-driven optimization paradigm requires unacceptable measurement cost to get the accuracy. In this paper, we mainly answer the following question: How does one get the interference accurately with rather low overhead? An efficient solution is proposed to tackle this. The solution reduces the measurement overhead and time cost through model-based derivation and scheduling. This solution could achieve both the reduction of measurement overhead and time cost. Moreover, this solution also provides the tool to control the tradeoff between accuracy and overhead. The experiments based on real testbed collected data traces are conducted to examine the performance of our solution. The results prove the efficiency of our solution and also give a hint about configuring the parameters in the algorithm.
